# McCleery Syndrome Caused by Pectoralis Minor Hypertrophy Treated with Multimodal Physical Therapy—A Case Report

**DOI:** 10.3390/jcm13102894

**Published:** 2024-05-14

**Authors:** Neven Starčević, Tadija Petrović, Tomislav Pavlović, Danijela Klarić, Dragan Primorac

**Affiliations:** 1 “St. Catherine” Specialty Hospital, 10000 Zagreb, Croatia; tadija.petrovic@svkatarina.hr (T.P.); tomislav.pavlovic@svkatarina.hr (T.P.); danijela.klaric@svkatarina.hr (D.K.); draganprimorac2@gmail.com (D.P.); 2 Medical School, University of Split, 21000 Split, Croatia; 3 Faculty of Medicine, Josip Juraj Strossmayer University of Osijek, 31000 Osijek, Croatia; 4 Faculty of Dental Medicine and Health, Josip Juraj Strossmayer University of Osijek, 31000 Osijek, Croatia; 5 Medical School, University of Rijeka, 51000 Rijeka, Croatia; 6 REGIOMED KLINIKEN, 96450 Coburg, Germany; 7 Eberly College of Science, The Pennsylvania State University, University Park, PA 16802, USA; 8 The Henry C. Lee College of Criminal Justice and Forensic Sciences, University of New Haven, West Haven, CT 06516, USA

**Keywords:** McCleery syndrome, axillary vein, brachial plexus, pectoral muscles, thoracic outlet syndrome, overhead athlete, pectoralis minor syndrome, pectoralis minor hypertrophy, physical therapy

## Abstract

We present a case of a healthy young male professional water polo player who presented with swelling and pain in the upper arm and elbow after vigorous exercise. Diagnostic workup included an MRI and dynamic duplex ultrasound, which revealed compression of the axillary vein by a hypertrophic pectoralis minor muscle without thrombosis, constituting McCleery syndrome. This is a rare entity within the multiple thoracic outlet syndrome aetiologies. Taking a detailed history and physical examination complemented with diagnostic imaging are vital to the diagnosis. Afterward, the patient was treated with multimodal physical therapy and fully recovered and even exceeded his previous training and play level.

## 1. Introduction

Thoracic outlet syndrome (TOS) refers to a diverse group of disorders and is defined as entrapment of neurovascular structures (the brachial plexus and the subclavian artery and vein), from their origin in the cervical region to the axilla. Since the anatomy of the region is complex, there are many potential sites of neurovascular entrapment, giving rise to varied clinical presentation and many names for the disorder (cervical rib syndrome, first thoracic rib syndrome, pectoralis minor syndrome, hyperabduction syndrome, and neurogenic pectoralis minor syndrome (NPMS)). It is usually divided into neurogenic TOS (>90% of all TOS cases) [1] and vascular TOS. The vascular type is further subdivided into arterial (<1% of all cases) and venous (5% of all cases). Typical symptoms include occipital headaches, pain, paraesthesia, weakness, numbness, swelling, coldness, tingling, and discoloration of the upper extremity [1]; it is important to note that the clinical presentation varies widely and clinical suspicion should be high when treating these patients, especially when symptoms are exacerbated by hair combing, carrying heavy objects, repetitive overhead arm maneuvers or sports activities or lifting heavy objects overhead (e.g., shelf stacking). A detailed anatomical classification of compression of the neurovascular structures includes three levels: the interscalene triangle, the costoclavicular space, and the pectoralis minor (PM) space. The interscalene triangle is formed by the anterior scalene muscle anteriorly, the middle scalene muscle posteriorly, and the superior aspect of the first rib. This is the first potential site of entrapment for the neurovascular bundle as the nerve roots exit their respective foramina coursing laterally and inferiorly, combining to become the superior, middle, and inferior trunks of the brachial plexus at the lateral border of the middle scalene muscle. The brachial plexus trunks are joined by the subclavian artery in a pass over the superior aspect of the first rib; at this level the subclavian vein is anterior to the anterior scalene muscle and therefore does not pass through the interscalene triangle. The following potential site of compression is the costoclavicular space bordered by the lateral edge of the first rib, the middle third of the posterior edge of the clavicle together with the subclavius muscle, and the scalene muscle insertions. The brachial plexus trunks divide into anterior and posterior divisions and the subclavian artery becomes the axillary artery. Travelling further inferiorly and laterally towards the axilla, the divisions again recombine to form the posterior, inferior, and lateral cord, named after their position relative to the axillary artery. The pectoralis minor (PM) muscle arises from the anterior side of the third to fifth rib and inserts on the medial and superior border of the coracoid process of the scapula; its function is to stabilize the scapula and draw it inferiorly and anteriorly. The PM space is just posterior to the PM muscle and its attachment at the coracoid process. The anatomical shape of the muscle creates a tunnel or passage under which the neurovascular bundle traverses, thus being the most distal potential entrapment site. At the lateral border of the PM muscle, the three cords divide and form the terminal branches of the brachial plexus. Compression in this space, an anatomical subdivision of TOS, is dubbed pectoralis minor syndrome (PMS) [1,2]. PMS refers to the site of compression and can be divided into neurogenic and vascular. Vascular PMS can affect the axillary artery or vein, while venous compression can be accompanied by axillary vein thrombosis (effort thrombosis or Paget–Schroetter disease). Compression of the axillary vein without thrombosis, i.e., non-thrombotic intermittent venous obstruction, is termed McCleery syndrome [3]. As stated above, venous TOS is uncommon and accounts for 5% of all TOS cases [1,4,5].

## 2. Case Presentation

After completing training sessions for the last two years, a 24-year-old male professional water polo player presented to our institution for swelling and weakness of the right upper arm and elbow without paraesthesias. The patient reported a sprain in the right shoulder 2.5 years ago that was neglected by the coaching staff due to the location of the pain being inferior to the clavicle, and training was continued.

Upon a physical exam, a kyphotic posture with forward scapular positioning was evident; the GIRD test was highly positive (internal rotation restriction at 40°), which is common in throwing athletes [6]. Also, the pectoralis major muscles were noted to be well-developed in contrast to the posterior scapular muscles. Fatigue was noted in 90° of abduction, and 90° of external rotation, and a feeling of filling in the arm; Roos and upper-limb tension tests [7] were positive on the right side, not uncommon in swimmers and water polo players [8].

**Figure 1:**
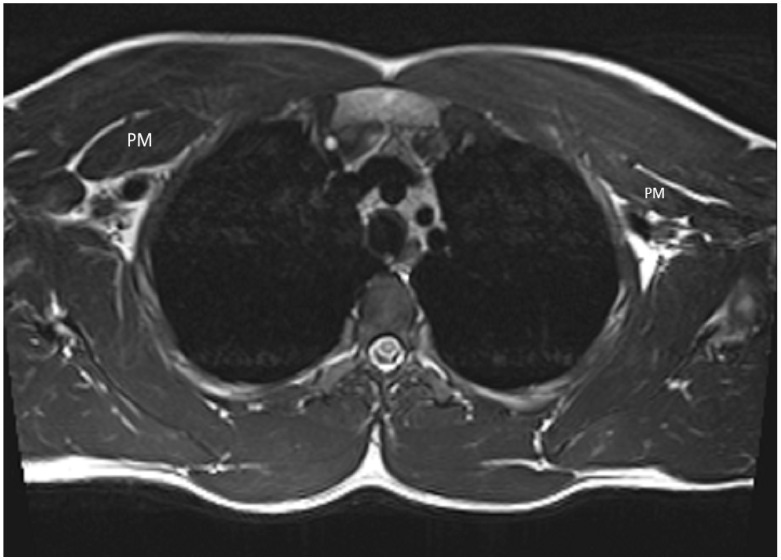
The MRI image shows a hypertrophic pectoralis minor muscle on the right side in contrast to the normal appearance on the left. (PM—pectoralis minor).

The work-up included an MRI of the cervical and thoracic spine, which showed syringomyelia without other pathology, and an MRI of the brachial plexus, which showed a hypertrophic pectoralis minor muscle on the right side (Figure 1).

### Nerve Conduction Studies Were Normal

**Figure 2:**
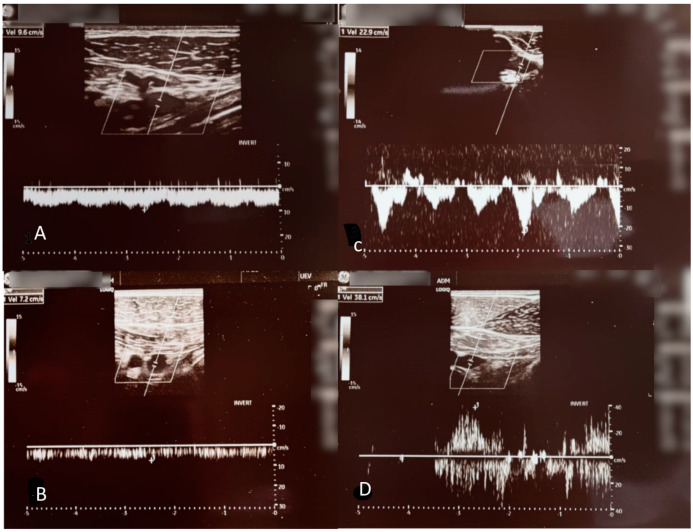
Dynamic duplex ultrasound shows attenuated blood flow in the right axillary vein (10 cm/s). (**A**) at rest and even slower flow after vigorous exercise (7 cm/s); (**B**) with little to no respiratory modulation, in contrast to the left side 23 cm/s at rest; (**C**) and 38 cm/s after vigorous exercise; (**D**) with normal respiratory modulation (in this image the velocities are shown inverted).

Duplex ultrasound venography was performed by a certified radiologist, which showed no evidence of thrombosis, excluding Paget–Schroetter syndrome. Still, a dynamic duplex ultrasound showed attenuated blood flow in the right axillary vein (10 cm/s) at rest and even slower flow after vigorous exercise (7 cm/s) in contrast to the left side, at 20 cm/s at rest and 40 cm/s after vigorous exercise (Figure 2).

Although the literature reports highly variable duplex ultrasound blood flow velocities in healthy volunteers [9], we believe the dynamic nature of the test and the observed velocity changes confirm the diagnosis of McCleery syndrome.

The patient was started on an intensive TOS-specific physical therapy regimen to correct posture and muscle imbalances and to stretch the pectoralis minor in order to relieve the pressure on the axillary vein [10].

The physical therapy regimen consisted of a manual muscle relaxation technique for the pectoralis minor and functional relaxation for both the pectoralis minor and major, along with post-isometric relaxation. The physical therapy method used was the WinteCare machine, which is based on TECAR technology. The therapeutic indication was to reduce the muscle tone of the hypertonic pectoralis minor muscle, and to serve as a starting point for the other manual procedures. 

To increase joint mobility and lessen the restricted internal rotation and dorsal glide, joint mobilization according to the Kaltenborn-Evjenth method was used, specifically the translators’ joint play.

Exercises with elastic bands were used to centralize the humerus, and they were also given to the patient for warmup before swimming/water polo training.

Proprioceptive-deep tendon reflex (P-DTR) is a therapeutic approach based on the principles of neurology and neurophysiology. The goal is to resolve musculoskeletal dysfunction by reestablishing sensory receptor signals and pathways potentially incorrectly integrated into the body.

In treating this patient, the P-DTR technique was used to find the stimuli that were sending aberrant information to the CNS and, with that, the correction of these stimuli. The stimuli that were sending inadequate information came from a combination of sources. Various thermal receptors, for example, a scar on the right shoulder, the problematic area, were created when the patient leaned on a lit cigarette. Various nociceptors are also elicited by tapping the palm on the right pectoralis muscle, or improper signal processing in the spinotectal tract (auditory signal), along with proprioceptors such as Golgi tendon organs and Pacini corpuscles. 

Moreover, deviations were determined in the optimal responses to mechanoreceptive stimuli using conscious and unconscious pathways such as the DCML and the spinocerebellar tract. These deviations were seen in the right acromioclavicular and sternoclavicular joint region, which are biomechanically dependent in specific shoulder movements, especially during horizontal abduction and adduction, flexion, and of the glenohumeral joint above 90 deg. The above-mentioned movements with elements of rotation are essential in the sport of water polo. The incorrect responses to these myotatic reflexes during anaerobic and aerobic stress were observed and corrected. 

**Figure 3:**
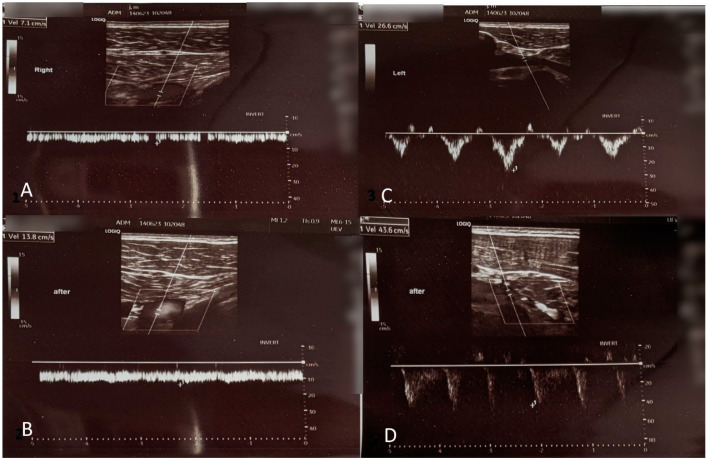
Dynamic duplex ultrasound shows attenuated blood flow in the right axillary vein 10 cm/s (**A**) at rest but in contrast to the findings before therapy, greater flow velocity after vigorous exercise 14 cm/s; (**B**) with little to no respiratory modulation, whereas on the left side 26 cm/s at rest; (**C**) and 43 cm/s after vigorous exercise; and (**D**) with normal respiratory modulation.

At three months follow-up, a Duplex ultrasound venography was performed by a certified radiologist, and no evidence of thrombosis were found. However, a dynamic duplex ultrasound still showed attenuated blood flow in the right axillary vein at 10 cm/s at rest, but in contrast to the findings before therapy, greater flow velocity after vigorous exercise at 14 cm/s in comparison to the left side at 26 cm/s at rest and 43 cm/s after vigorous exercise (Figure 3).

At six months follow-up, another Duplex ultrasound venography was performed by a certified radiologist, which showed no evidence of thrombosis, but dynamic duplex ultrasound showed further improvement of blood flow in the right axillary vein 15 cm/s at rest, 36 cm/s after vigorous exercise, in contrast to the left side 24 cm/s at rest and 45 cm/s after vigorous exercise.

**Figure 4:**
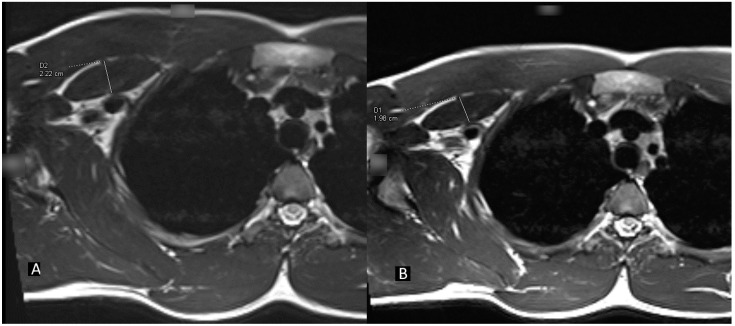
MRI image showing reduction of PM muscle thickness from 2.22 cm (**A**) to 1.98 cm (**B**), which represents a 10% reduction of thickness.

Follow-up MRIs of the shoulder girdle were performed at six and nine months since the initiation of physical therapy, as noted above, and showed moderate (10%) PM muscle thickness reduction (Figure 4).

After six months of multimodal physical therapy, the patient was able to return to the sport without any limitations that were present before therapy. Additionally, the patient was satisfied that surgery was not needed.

After nine months of multimodal physical therapy, the patient exceeded the previous level and volume of training without any symptoms.

At 12 months follow-up, the patient was completely asymptomatic during even the most intense training or match, and is a candidate for the water polo national team.

## 3. Discussion

Causes of compression in the pectoralis minor space vary; most are associated with traumatic incidents (after a motor vehicle collision or proximal humerus fracture), occupational factors (repetitive motion with the arm abducted) [11], and overhead athletes [2,12]. Usually, the patients complain of upper extremity pain and paresthesias, shoulder pain, arm cyanosis, and swelling [2], more often on the right side [13], which is hypothesized because most people are right-hand dominant. The symptoms arise due to the shortening of muscles or ligaments, scarring, or, as in our case, hypertrophy of the PM muscle.

It is important to differentiate between thrombotic (Paget–Schroetter syndrome), and non-thrombotic (McCleery syndrome) axillary vein obstruction as the treatments are vastly different. In this case, duplex ultrasound venography [11] was crucial to the diagnosis as it demonstrated attenuated blood flow in the right axillary vein compared to the left without thrombosis. Duplex ultrasound is a specialized interpretation of ultrasound waves that utilizes the Doppler shift property of sound waves to measure the speed of movement or flow in tissues. In clinical applications, it is usually used to measure flow velocity in a blood vessel. Since it does not use ionizing radiation, like classical venography, it can be performed multiple times. The other benefit of this test is the possibility of imaging both extremities quickly. In our case, we performed a Duplex ultrasound bilaterally, and both before and after vigorous exercise which is paramount to the diagnosis. At the first visit, the flow velocity in the right axillary vein was reduced compared to the left, which was symptom-free. After vigorous exercise performed during the visit, a Duplex ultrasound was again performed bilaterally and showed a further reduction in flow velocity in the right axillary vein, compared to the normally expected increase. An MRI showed pectoralis minor muscle thickening, all of which support the diagnosis of McCleery syndrome. During the physical therapy regimen, a follow-up duplex ultrasound venography showed, in contrast to the findings before therapy, greater flow velocity after vigorous exercise, while the baseline flow was still attenuated. By the end of treatment, the flow velocities were near the expected range for a healthy person.

The physical therapy regimen was aimed at correcting posture and muscle imbalances and stretching the pectoralis minor, in order to relieve the pressure on the axillary vein. Throwing and overhead sports dominantly use the anterior shoulder muscles and if not actively corrected lead to gross muscle imbalance as in our case. After posture correction, the next priority was PM muscle stretching and de-tonisation. TECAR therapy was started which employs high-frequency electromagnetic waves (0.3 to 1.2 MHz) and reduces activity-induced spasms and contractions. The Kaltenborn-Evjenth method is a complementary and alternative medicine (CAM) method based on gentle traction and passive motion aimed at reducing pain while increasing the affected joint range of motion. Lastly, the proprioceptive-deep tendon reflex (P-DTR) method was employed in order to further reduce the muscle tone of the PM and reestablish normal shoulder motion. While these physical therapy methods are far from having a robust evidence-based explanation in the literature, the combined effects on this patient, the symptoms, and axillary vein blood flow velocity was profound. Furthermore, the patient’s ability to train and compete at an even higher level then ever before speaks to the magnitude of effectiveness of treatment. We think that linking each specific treatment to the final outcome is not possible because the treatments and training schedule changes were concurrent, and their effects synergistic, so discerning individual contributions would therefore be conjecture and not scientific. 

In our opinion, electrophysiological studies are warranted to rule out associated neurological conditions or to confirm neurogenic TOS.

Another possible causative factor could be the aforementioned syringomyelia, but the authors do not believe that it contributed to the symptoms because of the excellent result of the conservative treatment and also due to the fact that the patient was complaining of pain and swelling in only the right arm, whereas we posit that the symptoms would be bilateral if the malformation of the cervical and thoracic spinal cord was the cause.

Initially, conservative treatment is indicated [14]; if the conservative treatments fail, surgical decompression by pectoralis minor tenotomy is warranted either by open [15] or arthroscopic means [16]. 

## 4. Conclusions

Compression of the axillary vein without thrombosis in the pectoralis minor space is a rare variant of TOS dubbed McCleery syndrome, presenting symptoms similar to subclavian vein obstruction. This rare condition is often neglected but should be included in the differential diagnosis of TOS if thrombosis is ruled out, especially in patients who frequently use their arms in the overhead position due to their occupation or participation in sports. This paper also highlights the efficacy of multimodal physical therapy in treating various compression syndromes. It should be the first line of therapy, with surgery reserved for patients who do not recover fully after physical therapy. In patients who do not respond to physical therapy at all, a different diagnosis should be sought. 

## Data Availability

All radiographic images are available upon request.
